# Subjective Psychophysical Experiences in the Course of Inflammatory Bowel Disease—A Comparative Analysis Based on the Polish Pediatric Crohn’s and Colitis Cohort (POCOCO)

**DOI:** 10.3390/ijerph18020784

**Published:** 2021-01-18

**Authors:** Aleksandra Glapa-Nowak, Anna Bukowska-Posadzy, Mariusz Szczepanik, Jarosław Kwiecień, Anna Szaflarska-Popławska, Barbara Iwańczak, Anna Flak-Wancerz, Łukasz Dembiński, Marcin Osiecki, Jarosław Kierkuś, Tomasz Banasiewicz, Harald Walach, Aleksandra Banaszkiewicz, Jarosław Walkowiak

**Affiliations:** 1 Department of Pediatric Gastroenterology and Metabolic Diseases, Poznań University of Medical Sciences, 60-572 Poznan, Poland; glapa@ump.edu.pl (A.G.-N.); anna.bukowska-posadzy@ump.edu.pl (A.B.-P.); mszczepanik@ump.edu.pl (M.S.); harald.walach@uni-wh.de (H.W.); 2 Department of Clinical Psychology, Poznan University of Medical Sciences, 60-812 Poznan, Poland; 3 Department of Pediatrics, Faculty of Medical Sciences in Zabrze, Medical University of Silesia, 40-055 Katowice, Poland; jkwiecien@sum.edu.pl; 4 Department of Pediatric Endoscopy and Gastrointestinal Function Testing, Collegium Medicum in Bydgoszcz, Nicolaus Copernicus University in Toruń, 85-067 Bydgoszcz, Poland; aszaflarska@wp.pl; 5 Department and Clinic of Pediatrics, Gastroenterology and Nutrition, Wroclaw Medical University, 50-369 Wroclaw, Poland; Barbara.iwanczak@umed.wroc.pl; 6 Department of Pediatrics, Faculty of Medical Sciences, Medical University of Silesia in Katowice, 40-752 Katowice, Poland; fitmed@wp.pl; 7 Department of Pediatric Gastroenterology and Nutrition, Medical University of Warsaw, 02-097 Warsaw, Poland; lukasz.dembinski@wum.edu.pl (Ł.D.); aleksandra.banaszkiewicz@wum.edu.pl (A.B.); 8 The Department of Gastroenterology, Hepatology, Feeding Disorders and Paediatrics, The Children’s Memorial Health Institute, 04-730 Warsaw, Poland; m.osiecki@ipczd.pl (M.O.); j.kierkus@med-net.pl (J.K.); 9 Chair and Department of General Surgery, Gastroenterological Surgical Oncology and Plastic Surgery, Poznań University of Medical Sciences, 60-355 Poznan, Poland; tbanasie@ump.edu.pl; 10 Department of Psychology, Witten/Herdecke University, D-58448 Witten, Germany; 11 Change Health Science Institute, D-13347 Berlin, Germany

**Keywords:** Crohn’s disease, ulcerative colitis, pain, anxiety, social activity

## Abstract

No gold standard is available to evaluate subjective psychophysical experiences in pediatric inflammatory bowel disease (IBD). We aimed to assess pain, anxiety, and limitations in social activities at diagnosis and the worst flare of the disease in relation to clinical expression, treatment and IBD severity. A total of 376 children completed the survey (Crohn’s disease (CD) n = 196; ulcerative colitis (UC) n = 180). The questionnaire included 12 questions regarding pain, anxiety, and social activity, all assessed at recruitment and retrospectively at diagnosis and worst flare using a numeric rating scale. Patients that had ever been treated with systemic glucocorticosteroids scored higher in pain (*p* < 0.001), anxiety (*p* = 0.015), and social activity domains (*p* < 0.016) at worst flare, and the answers correlated with the number of steroid courses (*p* < 0.0392). The perception of social activity limitations also correlated independently with the number of immunosuppressants (*p* < 0.0433) and biological agents (*p* < 0.0494). There was no difference in retrospective perception of pain, anxiety and social activity limitations between CD and UC patients at diagnosis and the worst flare. The level of limitations in social activity correlated with hospitalisations due to relapse, days spent in the hospital, number of relapses, and severe relapses with the strongest association of rho = 0.39 (*p* = 0.0004). Subjective and retrospective perception of pain, anxiety, and limitations in social activity differs depending on therapy, correlates with treatment modalities, and severity measures such as hospitalisations.

## 1. Introduction

Inflammatory bowel diseases (IBD) are chronic inflammatory conditions of the digestive tract inevitably associated with poor quality of life. Both of the major entities, Crohn’s disease (CD) and ulcerative colitis (UC) are characterised by unpredictable patterns of remissions and flares [1]. Symptoms frequently include abdominal pain, bloody diarrhoea, and fatigue, all of which hinder life activities, work, school, parenting, and social relationships [2,3,4,5].

Psychosocial symptoms, such as anxiety, often accompany the disease itself, and frequently, medications can also produce such symptoms [6,7]. Anxiety arises when we anticipate potential damage and could be defined as a feeling of unease, worry, and/or fear, that patients are unable to control [8]. IBD patients have higher rates of anxiety than the general population and compared to patients with other chronic diseases [9,10,11]. In adults with IBD, the rate of anxiety has been estimated between 29–35% during remission and as high as 80% during relapse [12]. Children with IBD also have higher anxiety compared with children with other chronic conditions [4]. Anxiety symptoms are correlated with disease activity, and medications such as glucocorticosteroids contribute an additional burden [5]. Therefore, an alert for warning symptoms may help indicate patients in need of adjunct therapy or psychosocial support.

Although the clinical burden of IBD is frequently measured with disease activity scales, such as the Pediatric Crohn’s Disease Activity Index (PCDAI) and Pediatric Ulcerative Colitis Activity Index (PUCAI) [13], these scales do not measure the patients’ subjective perception and experience of the illness. Several scales have been developed in adults, such as the health-related quality of life scale (HRQOL) [14,15], the short inflammatory bowel disease questionnaire (IBDQ) [16], orthe short health scale (SHS) [17]. However, no gold standard is available to date, and some are time-consuming to complete and evaluate [18]. In children, three scales are available to date (IMPACT, IMPACT-II, IMPACT-III), but none of them have been translated and validated in Polish [19]. Therefore, we combined essential factors to briefly and rapidly characterise the well-being in our cohort of children aged 3–18. The present study describes the patient-reported experiences for the global assessment of disease severity.

## 2. Materials and Methods

A total of four-hundred and six patients visited seven different centres (the Polish Pediatric Crohn’s and Colitis Cohort, POCOCO). Patients were recruited between April 2016 and March 2019, at the following academic departments:

- Department of Pediatric Gastroenterology and Metabolic Diseases, Poznań University of Medical Sciences;
- The Department of Gastroenterology, Hepatology, Feeding Disorders and Paediatrics; The Children’s Memorial Health Institute, Warsaw;
- Department of Pediatric Gastroenterology and Nutrition, Medical University of Warsaw;
- Department and Clinic of Pediatrics, Gastroenterology and Nutrition, Wroclaw Medical University;
- Department of Pediatrics, Faculty of Medical Sciences in Zabrze, Medical University of Silesia, Katowice;
- Department of Pediatrics, Faculty of Medical Sciences, Medical University of Silesia in Katowice;
- Department of Pediatric Endoscopy and Gastrointestinal Function Testing, Collegium Medicum in Bydgoszcz, Nicolaus Copernicus University in Toruń, Bydgoszcz.

A total of three-hundred and seventy-six patients from the POCOCO cohort agreed to fill in the questionnaire (92.6% response rate), and missing data were found in 25 out of 4512 items in total (0.55%). Exclusion and inclusion criteria were previously mentioned in [20]. Disease activity, nutritional status, and biochemical measurements at the time of questionnaire completion were assessed by consulting physicians blinded to the questionnaire results. Clinical relapse was defined as an intensification in symptoms and inflammatory markers that lead to an increase in hospitalization and disease management. The study obtained the approval of the Bioethical Committee at Poznan University of Medical Sciences (960/15 with the associated amendments).

The impact of IBD on lifestyle was assessed with the use of a customised questionnaire, including the measurement of three domains: pain, anxiety, and social activity (Figure S1). All patients were assessed at recruitment, diagnosis and worst flare. The social activity domain involved a question asking to what extent the disease limited participation in school/preschool activities and another one asked to what extent the disease limited their relationships with peers. The measurements were portrayed with a numerical rating scale (ranging from 0, “no effect”, to 10, “unimaginable effect”). The questionnaire was constructed by a clinical psychologist with over 30 years’ experience in the therapy of pediatric IBD. Cronbach’s α reliability coefficient for the whole scale across measurement times was α = 0.87, measured at different time points we obtained the following internal consistency measures: at the moment of diagnosis, α = 0.75; worst flare, α = 0.78; recruitment, α = 0.81.

The data were analysed using robust and non-parametric statistical methods. We reported median values and interquartile ranges [IQR] because they were less sensitive to outliers. Group differences were analysed using non-parametric Mann–Whitney U-tests and correlations were calculated as Spearman’s rank-coefficients, rho.

## 3. Results

A total of three-hundred and seventy-six patients completed the survey: 155 (41.2%) were female, and 196 (52.1%) had Crohn’s disease (Table 1). Disease localisation and behaviour are presented in the Table S1a,b.

The distribution of answers in the questionnaire are presented in Figure S2.

We did not observe differences in the distribution of answers regarding pain, anxiety, and social activity limitations depending on the disease category (UC/CD), at diagnosis (Table 2), or the worst flare (Table 3). At worst flare, all patients who received treatment (systemic glucocorticosteroids, immunosuppressive or biological treatment) scored higher at pain, anxiety and social activity limitations than patient without treatment (Table 3). We found that females scored higher in pain at diagnosis (females 7 (4–8) vs. males 6 (3–8), *p* = 0.0071) pain at worst flare (females 9 (7–10) vs. males 8 (5–9), *p* = 0.0094) and anxiety at worst flare (females 7 (5–8) vs. males 6 (4–8), *p* = 0.0263). The level of pain, anxiety and limitations in social activity at diagnosis and worst flare does not seem to be connected with the event of surgery (Table 2 and Table 3).

The higher the number of glucocorticosteroid courses, the higher the pain, both at diagnosis and at the worst flare, and anxiety at diagnosis (Table 4). The higher the number of immunosuppressants, the higher pain and anxiety ratings both at diagnosis and at the worst flare (Table 4). These treatments are also associated with limitations in peer relationships and relationships at school (Table 4). Patients that were older at first biological treatment experienced fewer limitations in peer relationships and school activities at the worst flare. A total of twenty patients (5.3%) took their first dose of both biological and immunosuppressive treatment simultaneously (within one month). There were no significant differences in pain, anxiety, and social limitations between them and other patients (neither at diagnosis nor the worst flare).

Patients with other concomitant diseases (e.g., celiac disease, bronchial asthma, obesity, gastroesophageal reflux disease, epilepsy, hypothyroidism) reported higher levels of anxiety at the worst flare (7 (5–9) vs. 7 (4–8); *p* = 0.0365) than patients without concomitant diseases. However, there were no differences for any of the studied experiences depending on extraintestinal IBD manifestations.

The limitations in social activity correlated significantly with hospitalisations due to relapse, days spent in the hospital, the number of relapses and severity of relapses (Table 5).

Some of the subjective psychological experiences correlate with Pediatric Ulcerative Colitis (PUCAI) and Pediatric Crohn’s Disease (PCDAI) activity indexes at diagnosis and at the worst flare, but only weakly (Table 6).

**Figure 1:**
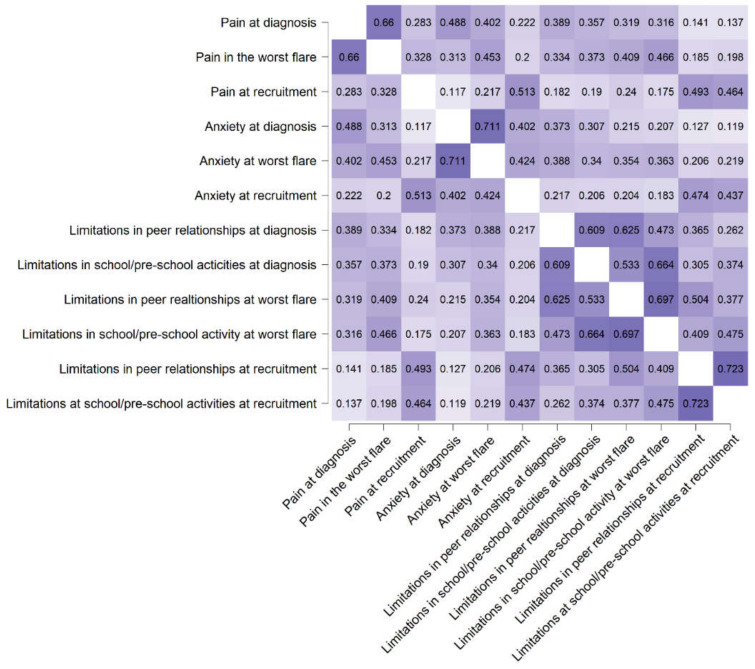
The relationships between measurements at recruitment, diagnosis and worst flare (Figure shows Spearman’s rho; darker colors indicate stronger relationships; *p* < 0.0103).

All the experiences studied (pain, anxiety, limitations at school, and in peer relationships) at diagnosis correlated strongly with their counterparts at the worst flare (Figure 1).

## 4. Discussion

To the best of our knowledge, the present study is the largest psychological description of Polish pediatric IBD in correlation with a comprehensive clinical picture. We identified no differences in retrospective perception of pain, anxiety, and limitations in social activity between CD and UC patients either at diagnosis or at the worst flare. Females reported more pain at diagnosis and the worst flare than males and also felt more anxious at the worst flare than males. Patients that had been ever treated with systemic glucocorticosteroids scored higher in pain, anxiety, and limitations in social activity domains at the worst flare. This was true also independently for immunosuppressive and biological treatments, but not for patients who underwent surgery. The level of limitation in social activities, such as school and peer relationships, correlated significantly but weakly with the number of hospitalisations due to relapse, days spent in hospital, the number of relapses and severity of relapses.

Numerous studies have shown that patients with IBD experience more anxiety than the healthy population [21,22,23,24]. Similarly, in Poland, most patients with IBD suffer a high level of anxiety [25]; however, this was found in small groups and adult cohorts. Andrzejewska et al. reported that the anxiety in the Polish population typically concerned cancer, having surgery or ostomy, and having access to a high-quality health service [26]. Several studies have shown that CD patients reported more anxiety than did patients with UC [27,28,29]; however, this finding is not consistent across studies [30]. In adults with IBD from Poland, no difference in anxiety has been reported, which is similar to our results [25,26]. There was no relationship between the type of disease and quality of life, either [26].

As previously shown, the amount of perceived physical symptoms is affected by neuroticism [31,32]. Morys et al. reported that 35% of adults with IBD in Poland presented high neuroticism [25]. Furthermore, they noticed a greater tendency for neuroticism in the UC group; however, the difference was not statistically significant [25].

Differences between men and women with IBD has been reported several times [33,34]. In our study, girls perceived more pain at diagnosis and worst flare than boys. They also felt more anxious at the worst flare than males. Hauser et al. showed that women with IBD experienced more anxiety and bowel symptoms than men [33]. The authors suggested that it is likely due to increased symptom perception in women [33].

Qualitative studies from several continents suggested that living with IBD negatively affects relationships and quality of life [35,36,37]. In a Polish adult group, the disease considerably hindered several activities, of which going to school or work (41.30%) scored the highest degree of impediment, followed by sleep (40.21%), physical activity (35.86%) and entertainment (34.78%) [26]. Data from a Canadian survey [37] has shown that the impact of the intestinal disease on relationships is severe; however, a Greek study ranks the impact as mild to moderate for most of the population [38]. In our study, the subjective sense of limitations in peer relationships at diagnosis and worst flare correlated positively with relapses, severe relapses, number of hospitalisations, and days spent in the hospital due to relapse. However, the strength of the associations in our study remained weak. Argyriou et al. showed that CD patients faced more limitations than UC, especially in relationships and work/school tasks [38]; however, we did not find differences between these two diseases.

We found that subjective pain, anxiety, and limitations in social activities correlated with treatment modalities. Loftus et al. found that steroids were a risk factor for developing anxiety disorders in young CD patients [24]. In our study, regardless of having steroid treatment later in life, patients scored similarly in the anxiety domain at diagnosis. Nevertheless, corticosteroids have been found to be one of the risk factors for psychological morbidity [39]. The same might be true for biological treatments and for immunosuppressive treatments. Given that children at a younger age seem to be more strongly affected, this might call for conservative approaches. Given the fact that lifestyle modifications have shown beneficial effects in adults with IBD [40,41,42], it might be worthwhile to consider conservative treatments, mind-body approaches and nutritional interventions for children in support of other treatment modalities, as well as psychosocial counselling.

The possible limitation of the present study is that subjective experiences of psychosocial issues were assessed retrospectively. Another limitation that might introduce bias is that tertiary centres, as the recruiting centres, tend to care for patients with severe disease. In Poland, however, clinical care of pediatric IBD patients is centralized. In addition, the studied cohort present rather moderate disease activity both at diagnosis and worst flare.

## 5. Conclusions

This study shows that subjective experiences such as pain, anxiety, and limitations in social life correlate with clinical features of pediatric IBD. The size of these retrospective associations is small, but not negligible, and the statistical association is clear. Thus, it is vital to consider mental health when making decisions about planning treatment in patients who suffer from IBD.

## Figures and Tables

**Table 1:** Demographic and clinical expression of Crohn’s disease and ulcerative colitis.

Variables Median (IQR) or n (%)	Crohn’s Disease n = 196	Ulcerative Colitis n = 180	*p*-Value
**Age [years]**			
at inclusion	15.0 (13.2–17.0)	15.2 (11.7–16.8)	0.0774
at diagnosis	12.4 (9.8–14.6)	12.1 (8.1–14.9)	0.4520
at worst flare	13.6 (11.5–15.9)	13.8 (10.2–15.9)	0.3694
**Duration of the disease (years)**	2.2 (0.7–4.2)	1.7 (0.4–3.7)	0.2430
**Sex**	75 (38.3) females	80 (44.4) females	0.2491
**Nutritional status**			
Weight at diagnosis (kg)	38.8 (27.0–51.0)	40.0 (28.8–54.0)	0.5455
Weight at diagnosis (z score)	−0.81 ([−1.38]–0.00)	−0.52 ([−1.14]–0.22)	0.0106
Height at diagnosis (cm)	150.5 (136.0–166.0)	151.5 (131.0–168.5)	0.7550
Height at diagnosis (z score)	−0.30 ([−1.20]–0.51)	0.11 ([−0.67]–0.86)	0.0042
Body mass index at diagnosis (kg/m^2^)	16.6 (14.6–18.4)	17.3 (15.5–19.1)	0.0407
Body mass index at diagnosis (z score)	−0.79 ([−1.45]–[−0.03])	−0.49 ([−1.02]–0.14)	0.0169
Albumin level at diagnosis (g/dL)	3.9 (3.5–4.3)	4.1 (3.7–4.41)	0.0028
**Parameter of inflammation**			
CRP at diagnosis (mg/L)	12.9 (2.0–30.3)	2.1 (0.5–9.5)	<0.0001
CRP at worst flare (mg/L)	13.0 (3.0–35.0)	2.7 (0.6–13.4)	<0.0001
**Disease activity scales**			
PCDAI/PUCAI at diagnosis	30 (23–48)	38 (30–53)	*
PCDAI/PUCAI at worst flare	40 (30–53)	56 (38–75)	*
**Treatment**			
Systemic glucocorticosteroids **	107 (54.6)	129 (71.7)	0.0007
Immunosuppressive treatment ***	153 (78.1)	103 (57.5)	<0.0001
Biological therapy ****	98 (50.0)	46 (25.6)	<0.0001
Operative treatment *****	24 (12.2)	3 (1.7)	<0.0001
**Subjective psychophysical experiences at recruitment**			
Pain	1 (0–2)	1 (0–3)	0.1528
Anxiety	2 (0–3)	2 (1–5)	0.0210
Limitations at school/preschool activities	1 (0–4)	2 (0–6)	0.0235
Limitations in peer relationships	0 (0–3)	1 (0–5)	0.0731

* incomparable due to dissimilarity of scales. ** Systemic glucocorticosteroid therapy included: methylprednisolone, prednisone, hydrocortisone. *** Immunosuppressive and anti-inflammatory agents included: azathioprine, methotrexate, mercaptopurine, cyclosporine, mycophenolate mofetil, tacrolimus. **** Biological agents included: infliximab, adalimumab, golimumab, vedolizumab. ***** Only surgery related to IBD-specific problems (e.g., colectomy, resection, fistula, perforation, abscess) was included. IQR, interquartile range; CRP, C-reactive protein (reference range 0–5 mg/L); PCDAI Pediatric Crohn’s Disease Activity Index; PUCAI, Pediatric Ulcerative Colitis Activity Index.

**Table 2:** Median and interquartile ranges of answers regarding pain, anxiety, and social activity at diagnosis depending on the treatment applied.

	Pain	*p*-Value	Anxiety	*p*-Value	Limitations in Peer Relationships	*p*-Value	Limitations in School Activities	*p*-Value
IBD								
UC	6 (4–8)	0.637	5 (3–8)	0.162	3 (0–7)	0.472	5 (3–8)	0.348
CD	6 (4–8)		5 (2–7)		3 (0–6)		5 (2–8)	
Systemic glucocorticosteroids								
Yes	7 (4–8)	**0.002**	5 (3–8)	0.105	4 (1–7)	**0.004**	5 (3–8)	0.057
No	6 (3–7)		5 (2–7)		2 (0–5)		4 (1–8)	
Immunosuppressive treatment								
Yes	7 (4–8)	**<0.001**	5 (3–8)	**0.009**	4 (1–7)	**<0.001**	5 (3–8)	0.103
No	5 (2–7)		4 (2–6)		2 (0–5)		4 (1–8)	
Biological therapy								
Yes	7 (4–8)	**0.015**	5 (2–8)	0.533	4 (1–7)	**0.007**	5 (2–9)	0.055
No	6 (3–8)		5 (3–7)		3 (0–5)		4 (2–8)	
Surgery *								
Yes	6 (4–7)	0.291	5 (3–6)	0.373	4 (1–6)	0.536	6 (3–10)	0.147
No	6 (4–8)		5 (3–8)		3 (0–6)		5 (2–8)	

* Only surgery related to IBD-specific problems (e.g., colectomy, resection, fistula, perforation, abscess) was included. Bold indicates statistically significant results (*p* < 0.05).

**Table 3:** Median and interquartile ranges of answers regarding pain, anxiety, and social activity at worst flare depending on the treatment applied.

	Pain	*p*-Value	Anxiety	*p*-Value	Limitations in Peer Relationships	*p*-Value	Limitations in School Activities	*p*-Value
IBD								
UC	8 (6–9)	0.661	7 (4–9)	0.095	5 (2–8)	0.556	8 (4–10)	0.091
CD	8 (5–9)		6 (4–8)		5 (1–8)		6 (3–9)	
Systemic glucocorticosteroids								
Yes	8 (6–10)	**<0.001**	7 (5–9)	**0.015**	6 (2–9)	**0.016**	8 (4–10)	**0.005**
No	8 (5–9)		6 (3–8)		4 (1–8)		6 (2–9)	
Immunosuppressive treatment								
Yes	8 (6–10)	**<0.001**	7 (5–9)	**0.031**	6 (2–8)	**0.003**	8 (4–10)	**0.003**
No	8 (4–9)		6 (3–8)		3 (0–8)		6 (1–9)	
Biological therapy								
Yes	9 (7–10)	**4 × 10^−5^**	7 (5–9)	**0.005**	7 (2–9)	**<0.001**	9 (5–10)	**0.019**
No	8 (5–9)		6 (4–8)		4 (1–8)		6 (2–9)	
Surgery *								
Yes	8 (7–10)	0.610	7 (5–8)	0.532	7 (4–9)	0.083	9 (4–10)	0.185
No	8 (6–9)		7 (4–8)		5 (1–8)		7 (3-10)	

* Only surgery related to IBD-specific problems (e.g., colectomy, resection, fistula, perforation, abscess) was included. Bold indicates statistically significant results (*p* < 0.05).

**Table 4:** Significant correlations of psychological experiences of patients with inflammatory bowel disease with parameters related to treatment.

Subjective Psychophysical Experiences	Treatment Parameter	*p*-Value	rho
Pain at diagnosis ^1^	Number of glucocorticosteroid courses	0.0005	0.18
	Number of immunosuppressants	0.0011	0.17
	Age at first immunosuppression	0.0146	−0.15
	Number of biological agents	0.0100	0.13
Pain at the worst flare ^2^	Number of glucocorticosteroid courses	0.0131	0.22
	Number of immunosuppressants	0.0005	0.18
	Number of biological agents	0.0229	0.21
Anxiety at diagnosis ^3^	Number of glucocorticosteroid courses	0.0392	0.11
	Number of immunosuppressants	0.0142	0.13
	Age at first immunosuppression	0.0268	−0.14
Anxiety at the worst flare ^4^	Number of immunosuppressants	0.0220	0.23
	Age at first immunosuppression	0.0085	−0.17
	Number of biological agents	0.0070	0.14
Limitations in peer relationships at diagnosis ^5^	Number of glucocorticosteroid courses	0.0002	0.20
	Number of immunosuppressants	0.0002	0.19
	Time to first immunosuppression	0.0455	−0.13
	Age at first immunosuppression	0.0238	−0.14
	Number of biological agents	0.0087	0.14
Limitations in peer relationships at the worst flare ^6^	Number of glucocorticosteroid courses	0.0220	0.23
	Number of biological agents	0.0005	0.18
	Age at first biological treatment	0.0271	−0.19
Limitations in school activities at diagnosis ^7^	Number of glucocorticosteroid courses	0.0100	0.13
	Number of immunosuppressants	0.0360	0.11
	Number of biological agents	0.0494	0.10
Limitations in school activities at the worst flare ^8^	Number of glucocorticosteroid courses	0.0026	0.26
	Number of immunosuppressants	0.0433	0.21
	Age at first immunosuppression	0.0247	−0.14
	Number of biological agents	0.0143	0.23
	Age at first biological treatment	0.0281	−0.19

Multiple linear regression model with the backward predictor entry using covariates from treatment parameter: ^1^ shows significant association only with age at first immunosuppression F (1244) = 4.15; *p* = 0.0428. ^2^ shows significant association with number of glucocorticosteroid courses (*p* = 0.0049), number of biological agents (*p* = 0.0078); F (1367) = 11.58; p=0.0090. ^3^ shows no significant association with any of the covatiates F (1243) = 3.86; *p* = 0.0506. ^4^ shows no significant association with any of the covariates F (1247) = 3.80; *p* = 0.0525. ^5^ shows significant association with number of glucocorticosteroid courses F (1245) = 11.94; *p* = 0.0006. ^6^ shows significant association with age at first biological treatment F (1137) = 6.93 *p* = 0.0095. ^7^ shows significant association with number of glucocorticosteroid courses F (1364) = 6.34; *p* = 0.0122. ^8^ shows significant association with age at first immunosuppression F (1126) = 9.33; *p* = 0.0028.

**Table 5:** The correlation of psychological experiences of patients with inflammatory bowel disease patients and disease severity.

Subjective Psychophysical Experiences	Severity Parameter Per 1 Year of The Disease	*p*-Value	rho
Pain at diagnosis	Hospitalisations for relapse	0.3182	0.07
	Days of hospitalisation for relapse	0.4678	0.05
	Relapses from diagnosis	0.0671	0.13
	Severe relapses from diagnosis	0.1278	0.11
Pain at the worst flare	Hospitalisations for relapse	0.0510	0.13
	Days of hospitalisation for relapse	**0.0243**	0.15
	Relapses from diagnosis	**0.0013**	0.22
	Severe relapses from diagnosis	**0.0123**	0.17
Anxiety at diagnosis	Hospitalisations for relapse	0.9622	0.00
	Days of hospitalisation for relapse	0.8922	–0.01
	Relapses from diagnosis	0.8685	–0.01
	Severe relapses from diagnosis	0.4897	0.05
Anxiety at the worst flare	Hospitalisations for relapse	0.3447	0.06
	Days of hospitalisation for relapse	**0.0412**	0.14
	Relapses from diagnosis	0.1706	0.09
	Severe relapses from diagnosis	**0.0097**	0.18
Limitations in peer relationships at diagnosis	Hospitalisations for relapse	**0.0327**	0.27
	Days of hospitalisation for relapse	**0.0047**	0.32
	Relapses from diagnosis	**0.0001**	0.26
	Severe relapses from diagnosis	**0.0016**	0.22
Limitations in peer relationships at the worst flare	Hospitalisations for relapse	**0.0254**	0.28
	Days of hospitalisation for relapse	**0.0019**	0.35
	Relapses from diagnosis	**0.0129**	0.29
	Severe relapses from diagnosis	**0.0048**	0.19
Limitations in school activities at diagnosis	Hospitalisations for relapse	**0.0172**	0.28
	Days of hospitalisation for relapse	**0.0165**	0.30
	Relapses from diagnosis	**0.0002**	0.25
	Severe relapses from diagnosis	0.0657	0.13
Limitations in school activities at the worst flare	Hospitalisations for relapse	**0.0049**	0.33
	Days of hospitalisation for relapse	**0.0004**	0.39
	Relapses from diagnosis	**0.0012**	0.37
	Severe relapses from diagnosis	**0.0078**	0.30

Bold indicates statistically significant results (*p* < 0.05).

**Table 6:** The correlation of psychological experiences of patients with inflammatory bowel disease patients and disease activity scales.

Subjective Psychophysical Experiences	Disease Activity Scale	*p*-Value	rho
Pain at diagnosis	PCDAI at worst flare	0.0003	0.28
Pain at worst flare	PCDAI at worst flare	0.0159	0.19
Limitations in peer relationships at diagnosis	PCDAI at diagnosis	0.0213	0.17
	PCDAI at worst flare	0.0109	0.20
Limitations in school activities at diagnosis	PCDAI at worst flare	0.0373	0.17
Limitations in school activities at worst flare	PCDAI at worst flare	0.0052	0.22
Pain at diagnosis	PUCAI at worst flare	0.0160	0.20
Pain at the worst flare	PUCAI at diagnosis	0.0122	0.20
	PUCAI at worst flare	0.0278	0.33
Anxiety at the worst flare	PUCAI at diagnosis	0.0210	0.18
	PUCAI at worst flare	0.0433	0.32
Limitations in peer relationships at diagnosis	PUCAI at diagnosis	0.0048	0.22
	PUCAI at worst flare	0.0425	0.17
Limitations in peer relationships at the worst flare	PUCAI at diagnosis	0.0137	0.19
	PUCAI at worst flare	0.0263	0.18
Limitations in school activities at diagnosis	PUCAI at diagnosis	0.0373	0.17
Limitations in school activities at the worst flare	PUCAI at diagnosis	0.0007	0.27
	PUCAI at worst flare	0.0001	0.31

PCDAI Pediatric Crohn’s Disease Activity Index; PUCAI, Pediatric Ulcerative Colitis Activity Index.

## Data Availability

Data available on request due to restrictions eg privacy or ethical.

## Supplementary Materials

The following are available online at https://www.mdpi.com/1660-4601/18/2/784/s1, Figure S1: Subjective psychophysical experiences in the course of chronic disease., Figure S2: Distribution plots of answers in psychologic assessment in regard to three domains: pain, anxiety, and social activity (school and peer relationships) measured at the time of recruitment, diagnosis and the worst flare. The measurements were portrayed with a visual analogue scale (0, no effect, 10, unimaginable effect)., Table S1a: Disease characteristics of the patients with Crohn’s disease enrolled in the study., Table S1b: Disease characteristics of the patients with ulcerative colitis enrolled in the study.

## References

[1] Ochsenkühn T., D’Haens G. (2011). Current misunderstandings in the management of ulcerative colitis. Gut.

[2] Devlen J., Beusterien K., Yen L., Ahmed A., Cheifetz A.S., Moss A.C. (2014). The burden of inflammatory bowel disease: A patient-reported qualitative analysis and development of a conceptual model. Inflamm. Bowel Dis..

[3] Dibley L., Norton C. (2013). Experiences of fecal incontinence in people with inflammatory bowel disease: Self-reported experiences among a community sample. Inflamm. Bowel Dis..

[4] Greenley R.N., Hommel K.A., Nebel J., Raboin T., Li S.-H., Simpson P., Mackner L. (2010). A meta-analytic review of the psychosocial adjustment of youth with inflammatory bowel disease. J. Pediatr. Psychol..

[5] Mackner L.M., Greenley R.N., Szigethy E., Herzer M., Deer K., Hommel K.A. (2013). Psychosocial issues in pediatric inflammatory bowel disease: Report of the North American Society for Pediatric Gastroenterology, Hepatology, and Nutrition. J. Pediatr. Gastroenterol. Nutr..

[6] Navabi S., Gorrepati V.S., Yadav S., Chintanaboina J., Maher S., Demuth P., Stern B., Stuart A., Tinsley A., Clarke K. (2018). Influences and Impact of Anxiety and Depression in the Setting of Inflammatory Bowel Disease. Inflamm. Bowel Dis..

[7] Choi K., Chun J., Han K., Park S., Soh H., Kim J., Lee J., Lee H.J., Im J.P., Kim J.S. (2019). Risk of Anxiety and Depression in Patients with Inflammatory Bowel Disease: A Nationwide, Population-Based Study. J. Clin. Med..

[8] Bannaga A.S., Selinger C.P. (2015). Inflammatory bowel disease and anxiety: Links, risks, and challenges faced. Clin. Exp. Gastroenterol..

[9] Katon W., Ciechanowski P. (2002). Impact of major depression on chronic medical illness. J. Psychosom. Res..

[10] Patten S.B., Beck C.A., Kassam A., Williams J.V.A., Barbui C., Metz L.M. (2005). Long-term medical conditions and major depression: Strength of association for specific conditions in the general population. Can. J. Psychiatry.

[11] Scott K.M., Bruffaerts R., Tsang A., Ormel J., Alonso J., Angermeyer M.C., Benjet C., Bromet E., de Girolamo G., de Graaf R. (2007). Depression-anxiety relationships with chronic physical conditions: Results from the World Mental Health Surveys. J. Affect. Disord..

[12] Nahon S., Lahmek P., Durance C., Olympie A., Lesgourgues B., Colombel J.F., Gendre J.P. (2012). Risk factors of anxiety and depression in inflammatory bowel disease. Inflamm. Bowel Dis..

[13] Best W.R., Becktel J.M., Singleton J.W., Kern F. (1976). Development of a Crohn’s disease activity index. National Cooperative Crohn’s Disease Study. Gastroenterology.

[14] Kappelman M.D., Long M.D., Martin C., DeWalt D.A., Kinneer P.M., Chen W., Lewis J.D., Sandler R.S. (2014). Evaluation of the patient-reported outcomes measurement information system in a large cohort of patients with inflammatory bowel diseases. Clin. Gastroenterol. Hepatol..

[15] Garrett J.W., Drossman D.A. (1990). Health status in inflammatory bowel disease. Biological and behavioral considerations. Gastroenterology.

[16] Irvine E.J., Zhou Q., Thompson A.K. (1996). The Short Inflammatory Bowel Disease Questionnaire: A quality of life instrument for community physicians managing inflammatory bowel disease. CCRPT Investigators. Canadian Crohn’s Relapse Prevention Trial. Am. J. Gastroenterol..

[17] Hjortswang H., Järnerot G., Curman B., Sandberg-Gertzén H., Tysk C., Blomberg B., Almer S., Ström M. (2006). The Short Health Scale: A valid measure of subjective health in ulcerative colitis. Scand. J. Gastroenterol..

[18] Guyatt G., Mitchell A., Irvine E.J., Singer J., Williams N., Goodacre R., Tompkins C. (1989). A new measure of health status for clinical trials in inflammatory bowel disease. Gastroenterology.

[19] Chen X.L., Zhong L.H., Wen Y., Liu T.W., Li X.Y., Hou Z.K., Hu Y., Mo C.W., Liu F.B. (2017). Inflammatory bowel disease-specific health-related quality of life instruments: A systematic review of measurement properties. Health Qual. Life Outcomes.

[20] Glapa-Nowak A., Szczepanik M., Kwiecień J., Szaflarska-Popławska A., Flak-Wancerz A., Iwańczak B., Osiecki M., Kierkuś J., Pytrus T., Lebensztejn D. (2020). Insolation and Disease Severity in Paediatric Inflammatory Bowel Disease-A Multi-Centre Cross-Sectional Study. J. Clin. Med..

[21] Byrne G., Rosenfeld G., Leung Y., Qian H., Raudzus J., Nunez C., Bressler B. (2017). Prevalence of Anxiety and Depression in Patients with Inflammatory Bowel Disease. Can. J. Gastroenterol. Hepatol..

[22] Graff L.A., Walker J.R., Bernstein C.N. (2009). Depression and anxiety in inflammatory bowel disease: A review of comorbidity and management. Inflamm. Bowel Dis..

[23] Abautret-Daly Á., Dempsey E., Parra-Blanco A., Medina C., Harkin A. (2018). Gut–brain actions underlying comorbid anxiety and depression associated with inflammatory bowel disease. Acta Neuropsychiatr..

[24] Loftus E.V., Guérin A., Yu A.P., Wu E.Q., Yang M., Chao J., Mulani P.M. (2011). Increased Risks of Developing Anxiety and Depression in Young Patients With Crohnʼs Disease. Am. J. Gastroenterol..

[25] Morys J.M., Kaczówka A., Jeżewska M. (2016). Assessment of selected psychological factors in patients with inflammatory bowel disease. Prz. Gastroenterol..

[26] Andrzejewska J., Talarska D., Michalak M., Linke K. (2009). Quality of life in patients with Crohn’s disease and ulcerative colitis. Comparative analysis. Gastroenterol. Rev. Prz. Gastroenterol..

[27] Nordin K., Påhlman L., Larsson K., Sundberg-Hjelm M., Lööf L. (2002). Health-Related Quality of Life and Psychological Distress in a Population-based Sample of Swedish Patients with Inflammatory Bowel Disease. Scand. J. Gastroenterol..

[28] Walker E.A., Roy-Byrne P.P., Katon W.J., Li L., Amos D., Jiranek G. (1990). Psychiatric illness and irritable bowel syndrome: A comparison with inflammatory bowel disease. AJP.

[29] Addolorato G., Capristo E., Stefanini G.F., Gasbarrini G. (1997). Inflammatory Bowel Disease: A Study of the Association between Anxiety and Depression, Physical Morbidity, and Nutritional Status. Scand. J. Gastroenterol..

[30] Häuser W., Janke K.-H., Klump B., Hinz A. (2011). Anxiety and depression in patients with inflammatory bowel disease: Comparisons with chronic liver disease patients and the general population. Inflamm. Bowel Dis..

[31] Richman L.S., Kubzansky L., Maselko J., Kawachi I., Choo P., Bauer M. (2005). Positive Emotion and Health: Going Beyond the Negative. Health Psychol..

[32] Ogińska-Bulik N., Juczyński Z. (2008). Personality traits conducive to somatic diseases—The role of type D. Psychoonkologia.

[33] Hauser G., Tkalcić M., Stimac D., Milić S., Sincić B.M. (2011). Gender related differences in quality of life and affective status in patients with inflammatory bowel disease. Coll. Antropol..

[34] Bielińska J., Liebert A., Lesiewska N., Bieliński M., Mieczkowski A., Sopońska-Brzoszczyk A., Brzoszczyk B., Długosz D., Guenter W., Borkowska A. (2017). Depressive and anxiety symptoms among patients with inflammatory bowel diseases. Med. Res. J..

[35] Lesage A.-C., Hagège H., Tucat G., Gendre J.-P. (2011). Results of a national survey on quality of life in inflammatory bowel diseases. Clin. Res. Hepatol. Gastroenterol..

[36] Muller K.R., Prosser R., Bampton P., Mountifield R., Andrews J.M. (2010). Female gender and surgery impair relationships, body image, and sexuality in inflammatory bowel disease. Inflamm. Bowel Dis..

[37] Becker H.M., Grigat D., Ghosh S., Kaplan G.G., Dieleman L., Wine E., Fedorak R.N., Fernandes A., Panaccione R., Barkema H.W. (2015). Living with inflammatory bowel disease: A Crohn’s and Colitis Canada survey. Can. J. Gastroenterol. Hepatol..

[38] Argyriou K., Kapsoritakis A., Oikonomou K., Manolakis A., Tsakiridou E., Potamianos S. (2017). Disability in Patients with Inflammatory Bowel Disease: Correlations with Quality of Life and Patient’s Characteristics. Can. J. Gastroenterol. Hepatol..

[39] Brooks A.J., Rowse G., Ryder A., Peach E.J., Corfe B.M., Lobo A.J. (2016). Systematic review: Psychological morbidity in young people with inflammatory bowel disease—Risk factors and impacts. Aliment. Pharmacol. Ther..

[40] Cramer H., Schäfer M., Schöls M., Köcke J., Elsenbruch S., Lauche R., Engler H., Dobos G., Langhorst J. (2017). Randomised clinical trial: Yoga vs written self-care advice for ulcerative colitis. Aliment. Pharmacol. Ther..

[41] Elsenbruch S., Langhorst J., Popkirowa K., Müller T., Luedtke R., Franken U., Paul A., Spahn G., Michalsen A., Janssen O.E. (2005). Effects of mind-body therapy on quality of life and neuroendocrine and cellular immune functions in patients with ulcerative colitis. Psychother. Psychosom..

[42] Langhorst J., Wulfert H., Lauche R., Klose P., Cramer H., Dobos G.J., Korzenik J. (2015). Systematic review of complementary and alternative medicine treatments in inflammatory bowel diseases. J. Crohns Colitis.

