# Supplementary material for: Subjective Psychophysical Experiences in the Course of Inflammatory Bowel Disease—A Comparative Analysis Based on the Polish Pediatric Crohn’s and Colitis Cohort (POCOCO)
Source: Int J Environ Res Public Health. 2021 Jan 18;18(2):784. doi: 10.3390/ijerph18020784 (PMC7831504; doi:10.3390/ijerph18020784)
Supplement: Supplementary file 1 [file ijerph-18-00784-s001.pdf]

Figure S1. Subjective psychophysical experiences in the course of chronic disease

I. Subjective pain

1. Using the 10-point scale below, indicate the intensity of your pain at diagnosis:

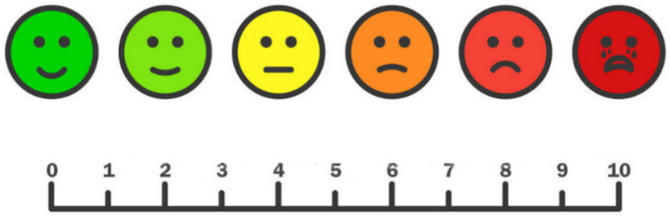

2. Using the 10-point scale below, indicate the intensity of pain you experienced during the time of the most intense symptoms.

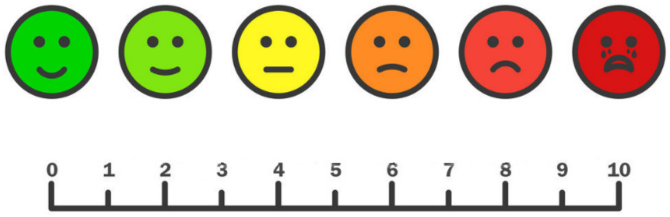

3. Using the 10-point scale below indicate the intensity of your current pain.

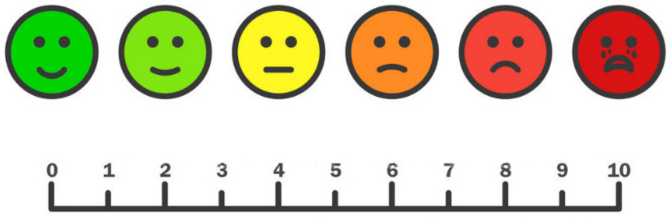

II. Subjective anxiety

1. On a 10-point scale, rate your level of anxiety related to the disease and treatment that you felt at the time of diagnosis (0, no anxiety, 10, maximum anxiety).

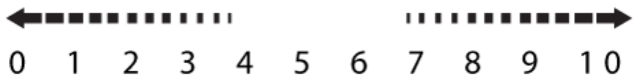

2. On a 10-point scale, indicate the level of your anxiety related to illness and treatment in the period of the highest intensity of disease symptoms (0, no anxiety, 10, maximum anxiety).

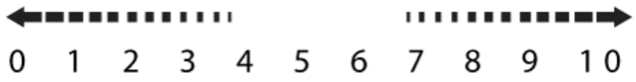

3. On a 10-point scale, indicate the level of anxiety you feel about the disease and the treatment you are currently experiencing (0, no anxiety, 10, maximum anxiety).

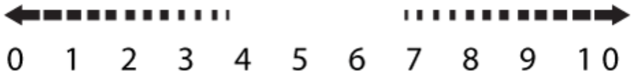

III. Limitations in social activity (social contacts, school/preschool)

1.

a) Indicate the extent to which the first symptoms (diagnosis period) of the disease limited your contacts with friends (0, had no effect, 10, had the maximum impact).

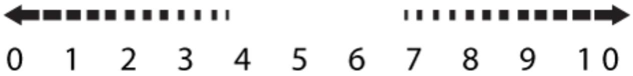

b) Indicate the extent to which the first symptoms (diagnosis period) of the disease limited your participation in school/preschool activities (0, had no effect, 10, had the maximum impact).

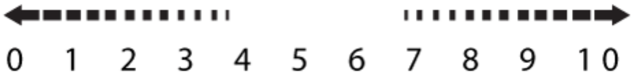

2.

a) Indicate to what extent the highest intensity of disease symptoms limited your contacts with friends (0, had no effect, 10, had the maximum impact).

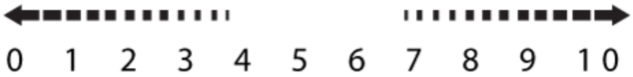

b) Indicate the extent to which the greatest intensity of disease symptoms limited your participation in school/preschool activities (0, had no effect, 10, had the maximum impact).

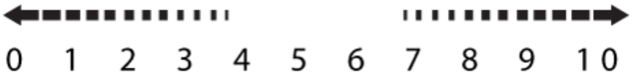

3.

a) Indicate the extent to which the disease limits your friends' contacts at present. (0, has no effect, 10, has maximum effect).

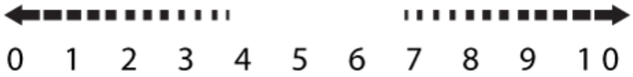

b) Indicate the extent to which the disease limits your participation in school/preschool activities currently. (0, has no effect, 10, has maximum effect).

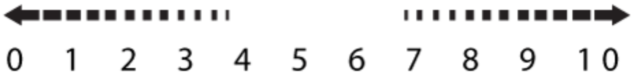

Figure S2. Distribution plots of answers in psychologic assessment in regard to three domains: pain, anxiety, and social activity (school and peer relationships) measured at the time of recruitment, diagnosis and the worst flare. The measurements were portrayed with a visual analogue scale (0, no effect, 10, unimaginable effect).

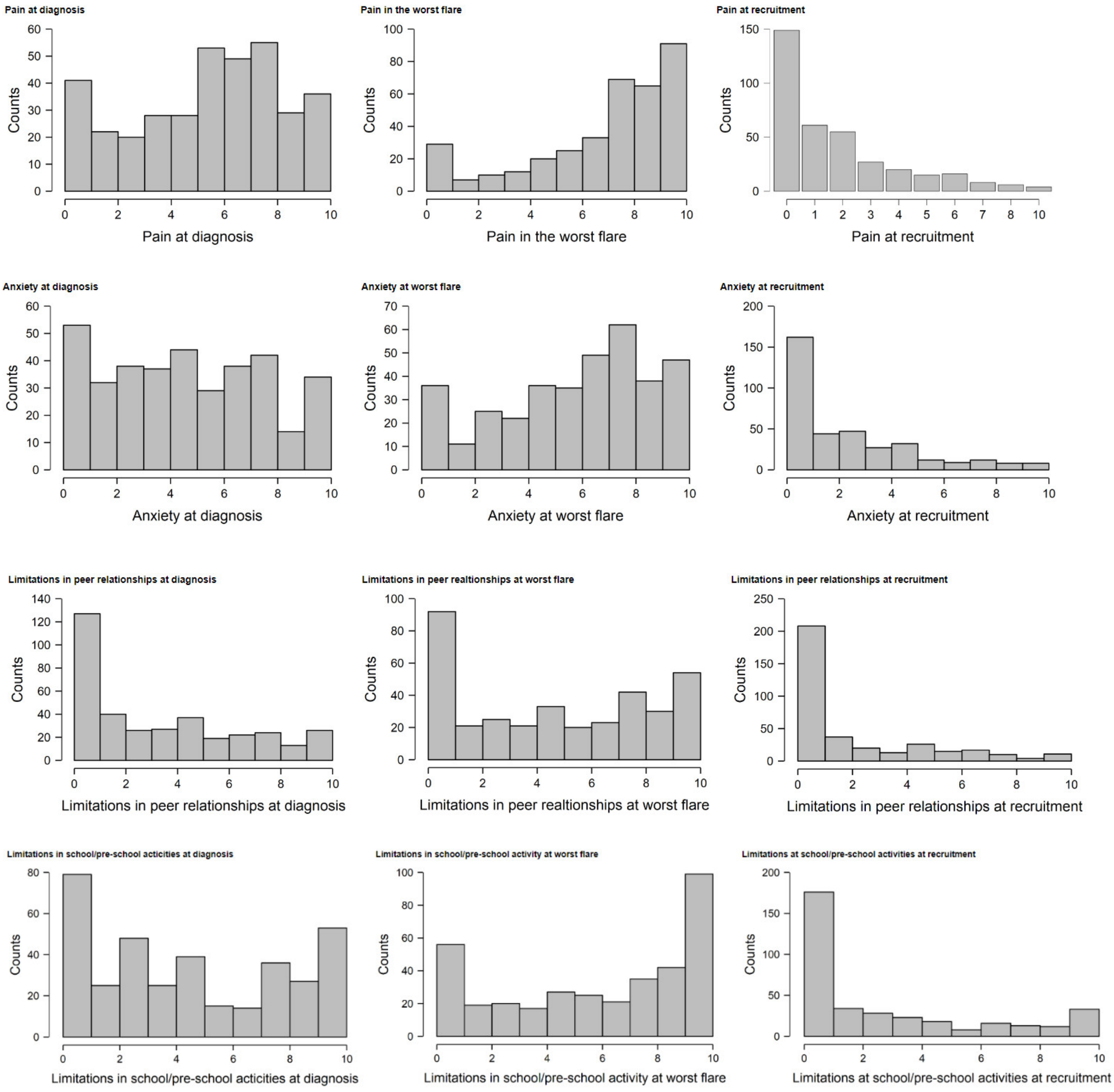

Pain at diagnosis
Counts
Pain at diagnosis
Pain in the worst flare
Counts
Pain in the worst flare
Pain at recruitment
Counts
Pain at recruitment
Anxiety at diagnosis
Counts
Anxiety at diagnosis
Anxiety at worst flare
Counts
Anxiety at worst flare
Anxiety at recruitment
Counts
Anxiety at recruitment
Limitations in peer relationships at diagnosis
Counts
Limitations in peer relationships at diagnosis
Limitations in peer realtionships at worst flare
Counts
Limitations in peer realtionships at worst flare
Limitations in peer relationships at recruitment
Counts
Limitations in peer relationships at recruitment
Limitations in school/pre-school acticities at diagnosis
Counts
Limitations in school/pre-school acticities at diagnosis
Limitations in school/pre-school activity at worst flare
Counts
Limitations in school/pre-school activity at worst flare
Limitations at school/pre-school activities at recruitment
Counts
Limitations at school/pre-school activities at recruitment

Table S1a. Disease characteristics of the patients with Crohn's disease enrolled in the study.

	Paris Classification	At diagnosis* n (%)	At worst flare** n (%)
Age	A1a: 0–10 years	51 (26.2)	20 (11.8)
	A1b: 10–16 years	133 (68.2)	130 (76.9)
	A2: 17–40 years	11 (5.6)	19 (11.3)
Location	L1: Ileal	49 (25.1)	38 (19.5)
	L2: Colonic	36 (18.5)	27 (13.9)
	L3: Ileocolonic	88 (45.1)	85 (43.6)
	L4a: upper disease proximal to the ligament of Treitz	22 (11.3)	18 (9.2)
	L4b: upper disease distal to the ligament of Treitz	7 (3.6)	8 (4.1)
Behaviour	B1: nonstricturing	138 (70.8)	112 (57.4)
	B2: structuring	14 (7.2)	19 (9.7)
	B3: penetrating	15 (7.7)	18 (9.2)
	B2B3: penetrating and stricturing	3 (1.5)	4 (2.1)
	P: perianal disease modifier	14 (7.2)	16 (8.2)
Growth	G0: no evidence of growth delay	138 (70.8)	117 (60.0)
	G1: growth delay	28 (14.4)	32 (16.4)

*out of total n=195; **out of total n=169

Table S1b. Disease characteristics of the patients with ulcerative colitis enrolled in the study.

	Paris Classification	At diagnosis n (%)	At worst flare n (%)
Extent	E1: Proctitis	17 (9.4)	7 (3.9)
	E2: Left-sided colitis	32 (17.8)	28 (15.6)
	E3: Extensive colitis	25 (13.9)	20 (11.1)
	E4: Pancolitis	88 (48.9)	80 (44.4)
Severity	S0: never severe	110 (61.1)	85 (47.2)
	S1: ever severe	37 (20.6)	48 (26.7)
